# Hints and Improvements in the Practice of Medicine, Surgery, and Pharmacy

**Published:** 1800-02

**Authors:** James Woodcock

**Affiliations:** Aldborough, near Aylsham, Norfolk


### HINTS AND IMPROVEMENTS

### IN THE PRACTICE OF

### MEDICINE, SURGERY, AND PHARMACY.

---

*To the Editors of the Medical and Phyſical Journal.*

GENTLEMEN,

AS the Phymoſis is a ſymptom that often occurs in conſequence of the venereal gonorhœa, and for the removal of which an operation becomes ſometimes neceſſary—if the following deviation from the uſual mode of relieving that complaint be thought deſerving a place in your Journal, you will oblige me by inſerting it therein.

The preputium being formed by a duplicature of the common integuments, connected together by a looſe cellular ſubſtance, it will be found in general (not always) to be the inner doubling of the ſkin, or that part next to the glans penis, which in moſt caſes forms the ſtricture in the phymoſis; the outward ſkin will commonly be found looſer, and to have but little concern in hindering the prepuce being drawn back; a diviſion of the inner duplicature only will be all that is neceſſary, in moſt caſes, to accompliſh the purpoſe of removing the ſtricture; and in all caſes will ſo far remove it, that in a few days, by proper treatment, the inflammation will ſubſide, ſo that the prepuce may be drawn back with eaſe.

So long ſince as the year 1769, when I was ſurgeon of the Prince of Wales Indiaman, Mr. ———, paſſenger in the ſaid ſhip, being in great pain from a phymoſis, with ſo much inflammation on the prepuce as to apprehend a ſloughing or mortification would be the conſequence, applied to me; after fomenting and ſyringing the parts frequently during the day, with ſuch medicines as I thought proper, the next day, he being no better, I repreſented to him the urgent neceſſity of removing the ſtricture, and explained to him the uſual operation on ſuch occaſions, and likewiſe a method I had before in contemplation; but how far it would ſucceed I was not able to tell, having never had an opportunity of performing it; but as this appeared appeared to him so much preferable to the other, he readily acquiesced, and I performed it in the following manner:

Having first drawn back the skin as far as I could, I gave it a snip with a pair of bent scissars, not more than about the eighth of an inch; I could now draw back the skin a considerable way further, so as to divide the inner doubling about a quarter of an inch more; this second snip gave so much liberty that I could now draw back and divide so much of the inner part as enabled me to get the whole prepuce over the glans, and both my patient and myself thought it a very judicious mode of operation, until I attempted to bring the skin forward again as I expected, but unfortunately was not able to do it. I was now fearful we were in a worse situation than before, as there was reason to expect a paryphymosis would be the consequence. As he felt little or no pain from girt or stricture above the glans, I put on a plaster of cooling cerate, fomented the part frequently, gave him cooling medicines, by which and the local bleeding, on the third day, the skin came forward, and with no more deformity at the edge of the prepuce than is often left after a well-performed operation for the hare-lip: I have performed this operation twice since; in both of which cases it fully answered my expectations.

Although in the first case a paraphymosis did not follow, it nevertheless gave me such alarm, that, in the other two, I took the precaution of dividing the inner skin higher up, (which I might have done in the first) and of not immediately retracting it over the glans; but brought it forward, till the inflammation subsided, and the prepuce would easily pass over the glans.

I would not wish it to be understood that I think this mode of operating applicable to every case of phymosis; many there are, where the integuments are so much thickened, and others where the connecting cellular membrane has acquired such a degree of adhesion by the inflammation, as not to allow of the necessary retraction.

Should this trifle meet with your approbation, I may send you some other (I believe, hitherto unthought of) medical hints.

I am, Gentlemen,

Your most obedient servant,

JAMES WOODCOCK.

*Aldborough, near Aylsham,*

*Norfolk, Jan.* 10, 1800.

*Account*

